# Advanced Materials, Machinability and Intelligent Manufacturing Systems

**DOI:** 10.3390/ma19071435

**Published:** 2026-04-03

**Authors:** Anna Burduk, Andre Batako, Anthony Xavior Michael, Suthep Butdee, Jose Machado, Kamil Krot

**Affiliations:** 1 Faculty of Mechanical Engineering, Wrocław University of Science and Technology, 27 Wybrzeże Stanisława Wyspiańskiego St., 50-370 Wroclaw, Poland; kamil.krot@pwr.edu.pl; 2 General Engineering Research Institute (GERI), Faculty of Engineering and Technology, Liverpool John Moores University, Byrom Street, Liverpool L3 3AF, UK; a.d.batako@ljmu.ac.uk; 3 School of Mechanical Engineering, Vellore Institute of Technology, Vellore 632014, India; 4 Division of Automation and Robotics Engineering, Department of Electrical and Telecommunication Engineering, Faculty of Engineering, Rajamangala University of Technology Krungthep, Bangkok 10120, Thailand; suthep.b@mail.rmutk.ac.th; 5 MEtRICs Research Centre, School of Engineering, University of Minho, Campus de Azurém, 4800-058 Guimarães, Portugal; jmachado@dem.uminho.pt

## 1. Introduction

The extremely rapid development of advanced materials and digital technologies has significantly changed modern industry [1,2]. The invention of new engineering materials, such as graphene, nano-diamond, nano-CBN, and their combination with other materials, has led to high-strength metal alloys and composite materials, and materials used in additive manufacturing have opened up a wide range of possibilities for designing and developing products with better functional performance [3,4]. New, advanced materials enable the engineering of high-strength, lightweight structures with increased resistance to extreme operating conditions, which is particularly important in sectors such as the aerospace, automotive, energy, and biomedical industries.

Advanced materials with high strength pose a series of technological challenges in manufacturing and production engineering [2,4,5,6,7]. Therefore, in recent years, research has increasingly focused on understanding the complex relationships between material properties and manufacturing processes. Modern, advanced materials are often characterised by complex microstructures, high hardness, or low thermal conductivity, which significantly hamper machining processes and lead to increased tool wear and deterioration of the surface layer [8]. Consequently, conventional manufacturing methods often require modification or replacement with new, advanced processing technologies. Furthermore, although intelligent methods are increasingly used in manufacturing environments, their integration with material selection and the optimisation of technological parameters remains limited [9,10,11]. Further research is needed on modelling and predicting complex phenomena occurring in manufacturing processes, such as springback, tool–workpiece interactions, and process stability, especially in the machining of advanced composite materials and the production of components with complex geometries. Therefore, researchers are increasingly integrating artificial intelligence (AI), machine learning (ML), and deep learning methods with manufacturing systems to optimise process parameters, predict defects, and increase process reliability [12,13,14]. These approaches enable data-driven design and production control by identifying complex relationships between process variables and material properties.

Another important research trend is the development of intelligent decision support systems in production [15,16,17]. The growing complexity of manufacturing processes requires advanced decision-making tools that support engineers in selecting appropriate materials, manufacturing technologies, and process parameters. Artificial intelligence methods, machine learning, fuzzy logic, and expert systems are being developed and increasingly utilised to improve decision-making processes in production engineering and production system management.

## 2. Research Issues and Contributions to This Special Issue

The ten articles in this Special Issue, “Advanced Materials, Machinability, and Intelligent Manufacturing Systems”, demonstrate how contemporary research integrates advanced materials science, manufacturing process modelling, and intelligent computational methods. These articles address key challenges related to processing advanced materials, increasing the efficiency of manufacturing processes, and implementing intelligent manufacturing systems capable of supporting next-generation industrial production.

One emerging research direction concerns the application of machine learning methods in advanced machining processes. In this context, Cortés-Mendoza et al. [Contribution 1] investigated the prediction of material removal rate and electrode wear in electrical discharge machining (EDM) using machine learning. EDM is widely used for machining hard and difficult-to-machine materials, particularly in the aerospace and tooling industries. The stochastic nature of the EDM process makes it difficult to predict machining results using conventional modelling approaches. The proposed machine learning framework enables the analysis of complex nonlinear relationships between process parameters and machining outcomes. The results demonstrate that machine learning techniques can significantly improve predictive capabilities and support EDM process optimisation in advanced manufacturing environments.

Another article addresses the issue of assessing manufacturability at the product design stage. Więcek et al. [Contribution 2] propose a method for assessing design decisions related to reducing material variability in single-item and small-batch production. This approach supports decision-making regarding material selection during product development and enables a reduction in production complexity by minimising the number of different materials used in manufacturing. This research contributes to improving production efficiency and sustainability, particularly in environments characterised by high product variability and low production volumes.

Balashov et al. [Contribution 3] present a method for optimising selective reinforcement in composite laminates, particularly for components manufactured using additive manufacturing technologies. The proposed optimisation approach enables efficient distribution of reinforcing material within composite structures while maintaining structural integrity and reducing weight. Such solutions are particularly important in industries where lightweight design is crucial, including the aerospace and transportation industries.

The paper by Kujawińska et al. [18] describes their work on the influence of glueing process parameters on the quality of adhesive joints in floorboards manufactured using innovative block glueing technology. The authors analysed the impact of pressing time and curing time for various adhesive systems. The results provide valuable information for optimising glueing technology and improving the reliability of engineered wood materials.

This Special Issue also includes research on advanced functional materials and sustainable thermal energy management technologies. Lv et al. [19] developed a scalable and cost-effective method for fabricating radiative cooling membranes using spray-coating technology. The proposed composite membrane demonstrates promising passive cooling properties and could contribute to the development of energy-efficient cooling systems for buildings and electronic devices. This research highlights the growing importance of advanced functional materials in addressing sustainability challenges.

A set of articles focused on modelling material behaviour during plastic forming processes, which is crucial in improving the predictability and efficiency of metal forming processes. Abd El-Aty and Shokry [20] conducted a comprehensive evaluation of phenomenological and physical constitutive models used to describe the behaviour of metallic materials during hot deformation. They compared different modelling approaches and assessed their predictive capabilities for various alloys under elevated temperature conditions. The results indicate that modified constitutive models can significantly improve the accuracy of numerical simulations used in metal forming processes. Alzahrani et al. [21] analysed the deformation behaviour of Al–Mg–Si alloys during hot deformation under various forming conditions. The authors analysed the flow stress behaviour using several modelling approaches, including crystalline plasticity models and modified constitutive equations. The obtained results provide valuable information on the complex deformation behaviour of aluminium alloys widely used in the automotive and aerospace industries and contribute to increasing the reliability of forming process simulations.

Another important research area represented in this Special Issue concerns the development of advanced metal processing technologies. Yang et al. [22] present a comprehensive review of ultrasonic liquid metal processing (UMP) technology in foundry processes. This technology is being recognised as a promising method for improving the microstructural refinement and mechanical properties of metallic materials. This article discusses recent developments, industrial applications, and future research directions for UMP technology, highlighting its potential for improving the sustainability and quality of foundry processes.

Skrzek et al. [23]. present the integration of intelligent decision support systems with manufacturing technologies. The authors developed an expert advisory system based on fuzzy logic to optimise the decision-making process regarding materials selection in additive manufacturing. The system supports engineers in assessing multiple criteria during material selection and helps reduce uncertainty in technological decision-making. The presented approach demonstrates the growing role of artificial intelligence methods in supporting engineering decisions in advanced manufacturing systems.

This Special Issue also includes research on precision forming technologies used in advanced energy systems. Su et al. [24] investigated the springback phenomenon during the hydroforming of microchannels in metal bipolar plates used in proton exchange membrane fuel cells. The authors analyse the effect of forming process parameters on the magnitude of springback and propose methods for improving dimensional accuracy in microforming processes. The results contribute to the development of manufacturing technologies for hydrogen energy systems and emphasise the importance of accurate modelling in microforming processes.

As Guest Editors, we express our sincere gratitude to all the authors who contributed their valuable research to this Special Issue. Equal thanks are extended to the reviewers for their constructive comments and valuable suggestions, which significantly improved the quality of the published papers.

We also express our heartfelt appreciation to the editorial team of *Materials* for their continuous support throughout the preparation and publication of this Special Issue.
